# Supplementary material for: Surveillance of a Pest Through a Public Health Information System: The Case of the Blackfly (Simulium erythrocephalum) in Zaragoza (Spain) during 2009–2015
Source: Int J Environ Res Public Health. 2020 May 25;17(10):3734. doi: 10.3390/ijerph17103734 (PMC7277163; doi:10.3390/ijerph17103734)
Supplement: Supplementary file 1 [file ijerph-17-03734-s001.pdf]

Supplementary Material

Surveillance of a Pest Through a Public Health Information System: The Case of the Blackfly (*Simulium erythrocephalum*) in Zaragoza (Spain) During 2009–2015

Ignacio Ruiz-Arrondo I [1,*], José A. Oteo [1], Javier Lucientes [2], Ana Muniesa [2] and Ignacio de Blas [2]

[1] Center for Rickettsiosis and Arthropod-Borne Diseases, Hospital San Pedro-CIBIR, Piqueras Street 98, 3rd floor, 26006, Logroño (La Rioja), Spain; jaoteo@riojasalud.es

[2] Faculty of Veterinary Sciences. Instituto Agroalimentario de Aragón (IA2), Miguel Servet Street 177, 50013, Universidad de Zaragoza, Zaragoza (Aragón), Spain; jlucien@unizar.es (J.L.); animuni@unizar.es (A.M.); deblas@unizar.es (I.d.B.)

* Correspondence: irarrondo@riojasalud.es; Tel.: +34-941278877

Received: 5 May 2020; Accepted: 22 May 2020; Published: date

Table S1. Basic Health Areas in the metropolitan area of Zaragoza.

ID	Basic Health Area (BHA)	Zone Type
1	Casablanca	Urban
2	Almozara	Urban
3	Fernando el Católico	Urban
4	Madre Vedruna-Miraflores	Urban
5	Rebolería	Urban
6	San José Centro	Urban
7	San Pablo	Urban
8	Actur Sur	Urban
9	Hernán Cortés	Urban
10	Las Fuentes Norte	Urban
11	San José Norte	Urban
12	Alfajarín	Rural
13	Avenida Cataluña	Urban
14	Delicias Sur	Urban
15	Valdefierro	Urban
16	Alagón	Rural
17	Casetas	Rural
18	María de Huerva	Rural
19	Villamayor	Rural
20	Parque Goya	Urban
21	Santa Isabel	Urban
22	Zuera	Rural
23	Fuentes de Ebro	Rural
24	Torre Ramona	Urban
25	Valdespartera	Urban
26	Independencia-Puerta del Carmen	Urban
27	Romareda-Seminario	Urban
28	Sagasta-Ruiseñores	Urban
29	San José Sur	Urban
30	Torrero-La Paz	Urban
31	Venecia	Urban
32	Universitas	Urban
33	Actur Oeste	Urban
34	Bombarda	Urban
35	Utebo	Rural
36	Delicias Norte	Urban
37	Miralbueno-Garrapinillos	Urban
38	Oliver	Urban
39	Actur Norte	Urban
40	Arrabal	Urban
41	Zalfonada	Urban
